# The interphalangeal angle as a novel radiological measurement tool for Morton’s neuroma – a matched case-control study

**DOI:** 10.1186/s13047-021-00502-7

**Published:** 2021-12-04

**Authors:** Martin Zaleski, Timo Tondelli, Sandro Hodel, Dominic Rigling, Stephan Wirth

**Affiliations:** Department of Orthopedics, Balgrist University Hospital, University of Zurich, Forchstrasse 340, 8008 Zurich, Switzerland

**Keywords:** Morton’s neuroma, Interphalangeal angle, Intermetatarsal angle, Forefoot disorders, Diagnosis, MRI, Dorsal-plantar X-ray

## Abstract

**Background:**

The aim of this retrospective study was to examine if a correlation between Morton’s Neuroma (MN) and an increased interphalangeal angle (IPA) or intermetatarsal angle (IMA) can be found in preoperative weightbearing dorsal-plantar X-rays of the foot.

**Methods:**

Forty-five patients with forty-nine MN in the interspaces 2/3 or 3/4 and 49 controls were recruited for this study. Every MN was matched with an asymptomatic control without history of metatarsalgia. The diagnosis was made by clinical examination, magnetic resonance imaging (MRI) and positive histopathology after operative resection. IMA 1/5, 2/3, 2/4, 2/5, 3/4 and IPA 2/3, 3/4 were measured for both groups.

**Results:**

The IPA 3/4 was significantly enlarged by 2.8 degrees (*p* < 0.001) with Area under the curve (AUC) 0.75 (*p* < 0.001), sensitivity of 73% and specificity of 67% in feet with MN compared to controls. The IMA 3/4 was significantly enlarged by 1 degree (*p* < 0.048) with AUC 0.64 (*p* < 0.031), sensitivity of 71% and specificity of 43% in feet with MN compared to controls. No difference between IMA 2/4, 2/5, 1/5 or correlation between IPA or IMA and the size of the MN in the MRI was found.

**Conclusion:**

The results confirm the clinical observation of an increased IPA in patients with MN. An increased IPA should therefore be considered in the diagnosis of MN.

**Supplementary Information:**

The online version contains supplementary material available at 10.1186/s13047-021-00502-7.

## Background

Morton’s Neuroma (MN) appears primarily in the female sex, with a female: male ratio of 4:1 [1]. Mean age at time of surgery is 50 years and it occurs bilaterally in 21%. It affects the third space in 66% of cases, the second in 32%, and the fourth in 2% [2]. The most common symptom is burning pain in the plantar aspect of the foot, located between the metatarsal heads, often radiating to the two corresponding toes. Hypesthesia and dysesthesia in the affected toes are often described [3–5]. Various causes of MN have already been discussed with regard to etiology like trauma [6], bursitis [6–8], anatomical variations [9–11], ankle equinus [12, 13], metatarsus proximus [7, 8], pronation [5, 9], and entrapment by the deep transverse metatarsal ligament [6, 14]. The MN is usually located proximal to the bifurcation in the digital nerves, just distal to the dorsal metatarsal transverse ligament (DMTL) and consists of a thickening of the interdigital nerve [15]. Macroscopically it has a fusiform configuration, a soft consistency and a white to yellowish appearance. Neural degeneration, epineural and endovascular hyalinization, and perineural fibrosis can be seen histologically [16, 17]. The diagnosis is usually made clinically. In addition, diagnostics are supplemented with imaging such as MRI or ultrasound [18]. Dorsal-plantar (DP) X-rays of the foot are essential to investigate other causes of metatarsalgia like tarsal–metatarsal joint pathologies, Freiberg’s disease, toe deformities or metatarsal–phalangeal instabilities [19, 20]. A simple radiographic measurement of digital divergence might be highly helpful to facilitate the diagnosis of a MN that sometimes can be difficult to distinguish from other forefoot disorders, especially when an MRI or an experienced ultrasound examiner is not available. An increased digital divergence in the intermetatarsal space affected by MN, that can be seen radiographically, was described before [21, 22]. Previous studies investigated a digital divergence radiographically caused by the MN, but failed to demonstrate a significant relationship [23, 24].

Reasons might be the measuring of subjects with MN in both intermetatarsal spaces 2/3 and 3/4 [24], no surgical histological confirmation of MN [24], the inclusion of patients with hallux valgus, cavus foot, hammer toes and arthritic deformities [23], different measuring methods [23] or the lack of a 1:1 matched case-control study design [23, 24]. In addition, none of these studies investigated the correlation between IPA and IMA using an MRI of the MN.

To overcome these limitations, a further analysis using an adequately powered case-control matching design is warranted.

By using weightbearing DP X-rays of the foot, we aimed to review this issue and analyze, if there is an association between MN and an increased interphalangeal angle (IPA) or intermetatarsal angle (IMA) in the affected interspace. Furthermore, a potential correlation of MN size and radiographic measurement in the MRI was evaluated.

## Methods

### Patients

Patients were selected which had a MN operatively resected in our clinic between 01/01/2016 and 12/31/2019 and met the following criteria: Inclusion criteria for MN patients were a minimum of 6-month history of neuroma symptoms, a clinical diagnosis of MN and confirmation thereof by MRI. The clinical diagnosis of MN was made by experienced senior physicians of our foot and ankle department and a senior musculoskeletal radiologist by MRI. All included MN were operatively resected and histologically examined in the institute of pathology. All resected samples were histologically diagnosed as MN by an experienced pathologist. Each foot with MN was matched by gender, age and the side of the foot with an asymptomatic control without history of metatarsalgia. The controls were patients of our foot and ankle outpatient clinic, who presented themselves with hindfoot pathologies like ankle distortion, ankle impingement or peroneal tendinopathy and received a weightbearing DP X-ray of the foot. The inclusion criteria for the matched control subjects was a negative history of MN or neuroma-like pain in the forefoot. Exclusion criteria for both MN and control groups were any previous surgery of the foot, any proximal nerve entrapment at the level of the ankle, knee, hip, or back, any history of significant trauma of the forefoot area, any difficulty in walking or standing, diabetes or systemic arthritis. Also excluded were patients with diagnosis of MN simultaneously in multiple webspaces in the same foot, as interdependencies between each other cannot be ruled out. In summary, the final sample consisted of 49 feet of 45 patients. Four patients had bilateral MN. There were 24 right feet and 25 left feet affected. The interspace 3/4 on the right side was affected in 19 feet, the interspace 2/3 on the right side was affected in 5 ft. The interspace 3/4 on the left side was affected in 20 feet, the interspace 2/3 on the left side was affected in 5 feet. The average age of patients with MN and control subjects was 50.7 (range 17.3–73.5) years and 50.6 (range 20.0–73.2) years, respectively. Participant consent was obtained and the study was approved by the cantonal ethics committee of Zurich, Switzerland (BASEC no.2019–01983). The patient characteristics are summarized in Table 1.

**Table 1:** Patient characteristics

	n
patients with MN	45
patients with bilateral MN	4
included feet with MN	49
right foot with MN	24
left foot with MN	25
3/4 interspace	39
2/3 interspace	10
controls	49
ethnicity of patients and controls	caucasian
mean age of patients with MN (sd)	50,7 (12,9) years
mean age of controls (sd)	50,6 (12,4) years
female patients with MN	36
female controls	39
male patients with MN	9
male controls	10

### Radiological measurements

**Fig. 1:**
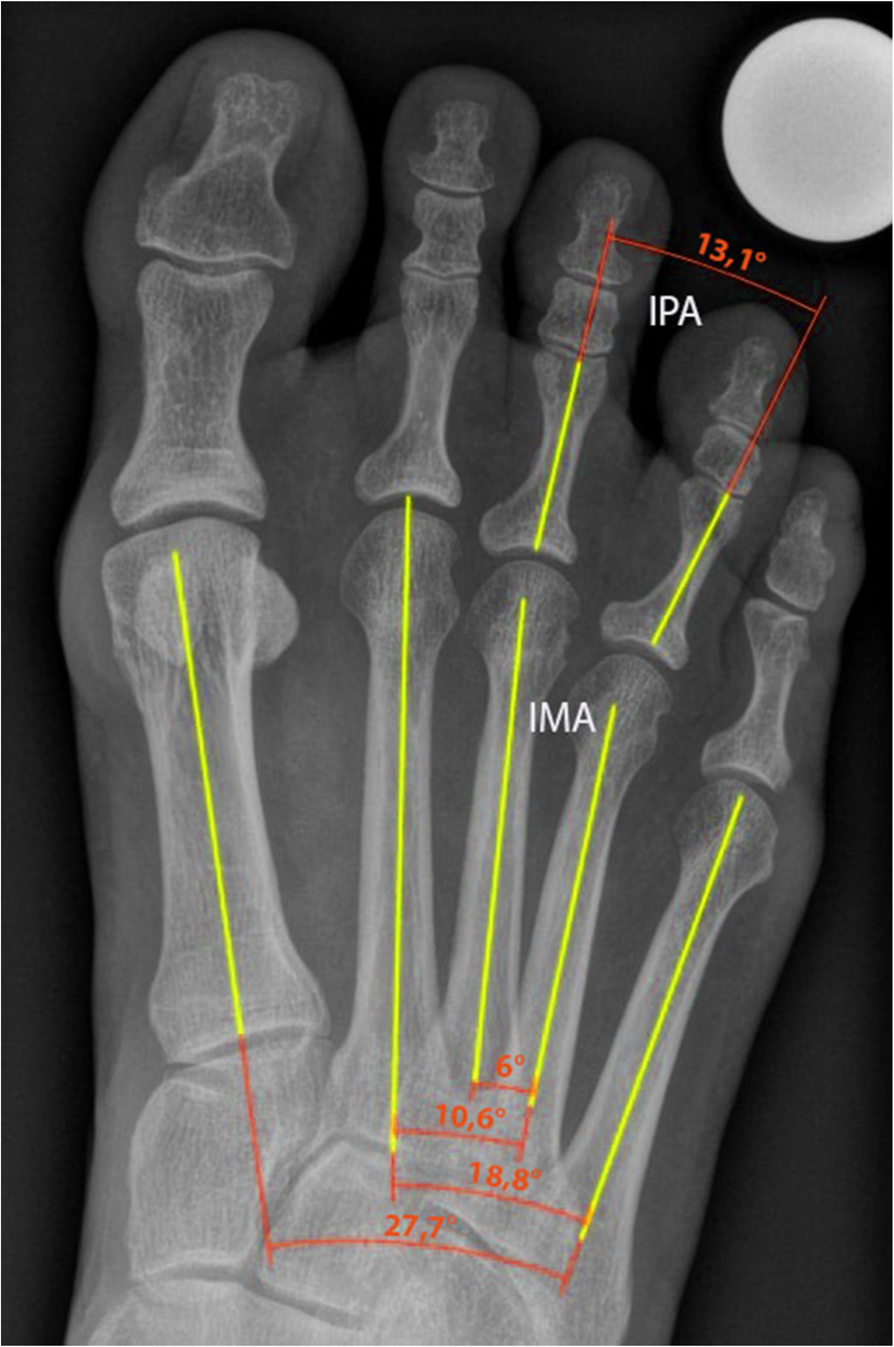
Radiological measurements. IPA = Interphalangeal angle; IMA = Intermetatarsal angle

Weightbearing DP X-rays of the foot of patients attending the foot and ankle department of our clinic were used in this study. The radiographs were performed preoperatively according to the same scheme of our radiological department. Two assessors performed the radiographic measurements for all patients and controls to check the interrater reliability. Intrarater reliability was assessed on radiographs from 10 randomly selected patients. These were reassessed 4 months after the initial measurements by one assessor. The radiographic measurements were performed via the orthopedic planning software mediCAD (mediCAD Hectec GmbH, Altdorf, Germany). After scaling every X-ray with a 25 mm planning ball, the center line of the diaphysis in the corresponding bone was measured with the function midline through 4 points: Two crosslines (with 2 points each) were made, one in the distal and the other one in the proximal diametaphysal junction in the proximal phalanx. Through these crosslines the measuring tool calculated a midline through the diaphysis. After that, the angle between the midlines of the diaphysis of the affected proximal phalanges was measured to assess the divergence of the proximal phalanges and defined as IPA. Additionally the IMA was measured in the same way (Fig. 1 Radiological measurements). The IMA 1/5, 2/3, 2/4, 2/5, 3/4 and the IPA 2/3, 3/4 were measured. If the lines diverged distally the value was positive, and -vice versa- negative, if the lines converged. The height and width of the MN were measured in the MRI in the coronal sectional view where it had its greatest extension.

### Statistics

The significance level was set at 0.05 and the results are reported as medians and range, unless stated otherwise. A paired t-test sample size estimation yielded a group size of 42 feet (alpha 0.05, power 0.8, minimally clinically important difference and standard deviation from previous research [24]). Case-control matching was performed according to side (exact matching), gender (exact matching) and age (median 0.8 years, maximum 2.7 years). Due to non-normal distributions of the interspace angles, non-parametric tests were used (Wilcoxon-signed-rank test). Inter- and intrarater reliability were measured by intraclass correlation coefficients (ICCs). Due to low numbers of affected feet, no separate receiver operating characteristic (ROC) analysis was performed for IPA 2/3 and IMA 2/3. For the remaining angles, the ROC curve, the Area under the curve (AUC) and its 95% confidence interval were calculated. The AUC was tested by a two-sided binomial z-test. The optimal cut-off value was determined by the minimum distance from the left-upper corner of the unit square. For multiple cut-off values, the diagnostic odds ratio was used as a second condition. This resulted in a unique cut-off value for every index based on the cut-off values, sensitivity, specificity and accuracy. *P*-Values were not adjusted for multiplicity with regard for sufficient power. The diagnostic odds-ratio was calculated. Association between MN coronal expansion (width and height) in the MRI and angles was assessed by Spearman’s rank correlation. Statistical analyses were computed using Stata/MP 15.1 software (StataCorp LP, College Station, TX, USA).

## Results

The radiographic measurements of weightbearing DP X-rays exhibited good to excellent interrater reliability with ICC between 0.79 and 0.96 (*p* < 0.001) and intrarater reliability with ICC between 0.96 and 0.99 (*p* < 0.001). Patients with MN had a significantly enlarged IPA 3/4 by 2.8 degrees (*p* < 0.001) compared to controls. IMA 3/4 was also significantly enlarged by 1.0 degree (*p* < 0.001) for MN patients compared to controls. There were no significant differences of the IPA 2/3 and IMA 2/3 between patients affected by MN and controls. Furthermore, we did not find a significant difference between IMA 2/4, 2/5, 1/5 and controls (Table 2).

**Table 2:** Radiographic measurements between groups

Interspace	MN (degrees)	Control (degrees)	Paired Diff.	*P*-value
IPA 3/4*	5.3 (−3.0; 14.1)	2.6 (− 5.7; 7.0)	2.8	*0.000
IMA 3/4*	6.2 (4.0; 10.2)	5.7 (1.4; 9.0)	1.0	*0.048
IPA 2/3	8.5 (3.9; 28.6)	4.4 (2.3; 10.5)	1.4	0.126
IMA 2/3	3.6 (0.4; 5.8)	3.0 (0.3; 5.4)	0.8	0.646
IMA 2/4	9.0 (3.8; 14.9)	8.6 (2.9; 13.1)	0.9	0.128
IMA 2/5	16.2 (7.5; 24.3)	17.0 (7.1; 24.3)	−0.3	0.874
IMA 1/5	25.0 (17.8; 32.3)	26.0 (14.9; 38.5)	0.4	0.925

Median and range (in brackets) in degrees. Paired difference denotes median of paired differences. *P*-values were calculated by Wilcoxon-signed rank test. *IPA* Interphalangeal angle; *IMA* Intermetatarsal angle

### Diagnostic characteristics

The diagnostic value of the IPA 3/4 yielded an AUC of 0.75 (*p* < 0.001), with a sensitivity of 73% and a specificity of 67% and the IMA 3/4 affected by MN compared to controls the AUC was 0.64 (*p* < 0.05), with a sensitivity of 71% and a specificity of 43%. The IMAs 2/4, 2/5 and 1/5 had no significant diagnostic value (Table 3). A separate calculation of the diagnostic performance for IPA 2/3 and IMA 2/3 was not feasible due to a too small sample size.

**Table 3:** Diagnostic performance

	IPA 3/4*	IMA 3/4*	IMA 2/4	IMA 2/5	IMA 1/5
Sensitivity	0.73	0.71	0.53	0.69	0.71
Specificity	0.67	0.43	0.55	0.39	0.41
Accuracy	0.70	0.57	0.54	0.54	0.56
OR	5.71	1.88	1.39	1.44	1.72
AUC	0.75	0.64	0.57	0.49	0.50
AUC CI 95	(0.65–0.85)	(0.52–0.75)	(0.46–0.66)	(0.39–0.59)	(0.4–0.6)
*P*-val (AUC = 0.5)	*0.000	*0.031	0.262	0.833	0.989

*OR* Diagnostic odds ratio, *AUC* Area under curve, *CI 95* 95% Confidence interval, *P-val* p-value; P-val (AUC = 0.5) denotes *p*-value of each AUC tested against 0.5, binomial z-test; *IPA* Interphalangeal angle, *IMA* Intermetatarsal angle

### MRI size correlation

The MN had a width of 1.0 mm (0.0; 1.0 mm) and a height of 6.4 mm (3.4; 11.5 mm) in the MRI’s coronal plane. The correlation between IPA (2/3 and 3/4) and MN width and height was non-significant and low at 0.15 (*p* = 0.316) and 0.08 (*p* = 0.574), respectively. This was also true for the correlations between the affected IMA and MN width and height which were 0.18 (*p* = 0.221) and 0.20 (*p* = 0.161). We found no significant correlation between MN size and IMA 2/4, 2/5 and 1/5.

## Discussion

The most important finding of our study is that an increased IPA 3/4 was associated with the presence of MN in the corresponding web space and may raise suspicion for the presence of a MN and facilitate clinical diagnosis.

The presence of an increased IPA 3/4 and IMA 3/4 can aid to support clinical diagnosis of a suspected MN in the corresponding webspace. As the diagnostic performance was only of moderate nature, it should be considered as an additional radiographic support, rather than a reliable screening or confirmation tool for the presence of a MN. This is especially of clinical relevance, if MRI diagnostics or an experienced ultrasound investigator is unavailable.

Previous studies failed to demonstrate differences in weightbearing DP X-rays between patients affected by MN and controls [23, 24]. These contrasting findings, most likely occurred due to the inclusion of combined MN of adjacent intermetatarsal spaces 2/3 and 3/4 [24], the inclusion of patients with different foot deformities and the use of different measuring methods [23].

To overcome this potential bias, we measured the divergence angles of the second and third interspaces separately. However, it should be noted that isolated MN 2/3 occurs less frequent and therefore the sample size of this subgroup is relatively small [2]. Our findings of an increased IPA and IMA support the theory that the MN exerts pressure on the distal metatarsals and the proximal phalanges in the corresponding interspaces [21–23]. Surprisingly, we did not find a correlation of the size of the MN in the MRI for the increased IPA 3/4.

Our study design differs from most previous studies in other respects: In order to control for potential confounding variables and to minimize bias, each patient with a MN was matched to a control by gender, age and the side of the foot. In addition to preoperative MRI, the presence of a MN was confirmed by an independent histopathological examination after resection, which allowed the diagnostic confirmation of a MN with great certainty. To minimize confounding factors that could influence the IPA or IMA, we applied strict exclusion criteria like previous surgery of the forefoot, any proximal nerve entrapment, trauma of the forefoot, diabetes or arthritis. The described x-ray measurements demonstrated good to excellent inter- and intrarater reliability.

Like Park et al., we could not demonstrate a significant correlation of the width of the forefoot in patients with MN compared to controls [25]. Neither between IMA 1/5, nor 2/4 or 2/5 demonstrated an increased width of the forefoot in MN patients.

When comparing the diagnostic performance of an increased IPA in X-ray to MRI and ultrasound, a superior sensitivity of 93 and 90% respectively could be demonstrated for the later two. The reported specificity of 68% of MRI was similar to our findings, whereas the specificity of ultrasound with 88% also demonstrated superiority [18]. Nevertheless, advantages of the presented measurement include the good to excellent reliability of a simple X-ray measurement, its all-time availability and low costs compared to MRI. Moreover, ultrasound diagnostics are often dependent on the experience of the investigator.

Overall, the findings in this study provide additional information in the diagnosis of MN using simple weightbearing DP X-rays of the foot without the use of advanced imaging. The use of weightbearing DP X-rays of the foot are part of the state-of the art diagnostics to rule out other causes of metatarsalgia in patients with suspected MN [19, 20].

### Limitations

Several limitations should be considered when interpreting our findings. The results of this study apply to patients who were scheduled for MN excision, which could have favored a selection bias towards bigger MN. Not taken into account were ligament insufficiencies of the MTP joint which might be also a reason for an increased IPA. It should be mentioned that the diagnostic performance of the significantly enlarged IMA 3/4 is moderate. Four patients had a bilateral MN and it is not taken statistically in account, that the right and left foot are highly correlated within the same person. Another statistic limitation is a joint probability of a Type I error higher than 0.05 due to the lack of multiplicity correction.

## Conclusion

The results confirm the clinical observation of an increased IPA in patients with MN. An increased IPA should therefore be considered in the diagnosis of MN.

## Supplementary Information

**Additional file 1.**

## Data Availability

Patients were selected which had a MN operatively resected in our clinic between 01/01/2016 and 12/31/2019. The controls were patients of our foot and ankle outpatient clinic, who presented themselves with hindfoot pathologies between 01/01/2016 and 12/31/2019. The datasets of the patients and controls generated and analysed during the current study are not publicly available, to not compromise individual privacy. The data is available from the corresponding author on reasonable request.

## Abbreviations

IPA: Interphalangeal angle
MN: Morton’s Neuroma
IMA: Intermetatarsal angle
MRI: Magnetic resonance imaging
AUC: Area under the curve
DMTL: Dorsal metatarsal transverse ligament
DP: Dorsal-plantar
ICCs: Intraclass correlation coefficients
ROC: Receiver operating characteristic
