# Supplementary material for: The interphalangeal angle as a novel radiological measurement tool for Morton’s neuroma – a matched case-control study
Source: J Foot Ankle Res. 2021 Dec 4;14:62. doi: 10.1186/s13047-021-00502-7 (PMC8642935; doi:10.1186/s13047-021-00502-7)
Supplement: Supplementary file 1 — Additional file 1. [file 13047_2021_502_MOESM1_ESM.docx]

Reviewer #1:

Background - there have been no changes made to the background section to address my comments (nor the background-related comments from the other reviewers). The background still lacks sufficient justification for the current study and requires further clarification around the relationship between angular radiographic measurements and morton's neuroma.

Answer: We added a justification and a clarification around the relationship between angular radiographic measurements and morton's neuroma in the background section (line 60-75) and we hope that it meets the reviewer´s expectations. Your constructive criticism regarding the background was fully justified. We hope the background now better describes the reasons that led us to this study.

Methods - thank you for addressing the confusion re: foot vs. patients. An additional table has been added in the middle of the manuscript, but there is no reference to this table in the text.

Answer: A reference has been made (line 117).

Statistics - thank you for providing more information re: the sample size calculation. If you are not adjusting your p-values for multiplicity you need to justify the reason for this decision in your methods section and address this in the discussion as a limitation along with the other statistical limitations present in your manuscript.

Answer:

Thank you for your comments. As requested we added a justification not correcting for multiplicity to the methods (line 167-168) and limitation section (line 275-277).

Table 2 - thank you for providing the confidence intervals. It makes interpretation of the results easier.

Discussion - the discussion is still quite disjointed and difficult to read (it is currently written as a single 3-page-long paragraph). You may want to consider separating out the main points into different paragraphs.

ANSWER: Thank you for your constructive criticism regarding the discussion. We changed it and included a separate limitation section. We hope its better now and easier to read.

@Reviewer #1: Thank you very much for your comments. They helped to improve the manuscript.

Reviewer #2:

Thank you for the amendments to the manuscript.

Overall, it reads better with key emphasis of the findings, however some of the phrases (additions in red) - for example the latter part of the discussion section on pages 10 and 11 (lines 250 - 256 could be modified for grammar and flow.

ANSWER: Thank you for your constructive criticism regarding the discussion. We modified it for grammar and flow and hope it is better to read now.

@Reviewer #2: Thank you very much for your comments. They helped a to improve the manuscript.

Reviewer #3:

Thank you for your revisions which has improved the manuscript.

Two minor points:

- the table of patient characteristics needs refining to make it a little easier to read and analyse (maybe consider adding another column for controls)

ANSWER: The spacing in the table has been adjusted to make it easier to read. A reference has been made (line 117).

-the green text in the figure is still difficult to read

ANSWER: We changed the figure and hope its good readable now.

@Reviewer #3: Thank you very much for your comments. They helped a to improve the manuscript.
